# Rapid Proteomic Characterization of Bacteriocin-Producing *Enterococcus faecium* Strains from Foodstuffs

**DOI:** 10.3390/ijms232213830

**Published:** 2022-11-10

**Authors:** Marcos Quintela-Baluja, Kelly Jobling, David W. Graham, Shamas Tabraiz, Burhan Shamurad, Mohamed Alnakip, Karola Böhme, Jorge Barros-Velázquez, Mónica Carrera, Pilar Calo-Mata

**Affiliations:** 1 Department of Analytical Chemistry, Nutrition and Food Science, School of Veterinary Sciences, University of Santiago de Compostela, Campus Lugo, 27002 Lugo, Spain; 2 School of Engineering, Newcastle University, Newcastle upon Tyne NE1 7RU, UK; 3 School of Natural and Applied Sciences, Canterbury Christ Church University, Canterbury CT1 1QU, UK; 4 UDE Engineering Ltd., Newcastle upon Tyne NE4 5AJ, UK; 5 Department of Food Control, Faculty of Veterinary Medicine, Zagazig University, Zagazig 44519, Egypt; 6 Department of Food Technology, Spanish National Research Council (CSIC), Marine Research Institute (IIM), 36208 Vigo, Spain

**Keywords:** food safety, proteomics, *Enterococcus faecium*, probiotics, bacteriocins, TimsTOF

## Abstract

Enterococcus belongs to a group of microorganisms known as lactic acid bacteria (LAB), which constitute a broad heterogeneous group of generally food-grade microorganisms historically used in food preservation. Enterococci live as commensals of the gastrointestinal tract of warm-blooded animals, although they also are present in food of animal origin (milk, cheese, fermented sausages), vegetables, and plant materials because of their ability to survive heat treatments and adverse environmental conditions. The biotechnological traits of enterococci can be applied in the food industry; however, the emergence of enterococci as a cause of nosocomial infections makes their food status uncertain. Recent advances in high-throughput sequencing allow the subtyping of bacterial pathogens, but it cannot reflect the temporal dynamics and functional activities of microbiomes or bacterial isolates. Moreover, genetic analysis is based on sequence homologies, inferring functions from databases. Here, we used an end-to-end proteomic workflow to rapidly characterize two bacteriocin-producing *Enterococcus faecium* (*Efm*) strains. The proteome analysis was performed with liquid chromatography coupled to a trapped ion mobility spectrometry-time-of-flight mass spectrometry instrument (TimsTOF) for high-throughput and high-resolution characterization of bacterial proteins. Thus, we identified almost half of the proteins predicted in the bacterial genomes (>1100 unique proteins per isolate), including quantifying proteins conferring resistance to antibiotics, heavy metals, virulence factors, and bacteriocins. The obtained proteomes were annotated according to function, resulting in 22 complete KEGG metabolic pathway modules for both strains. The workflow used here successfully characterized these bacterial isolates and showed great promise for determining and optimizing the bioengineering and biotechnology properties of other LAB strains in the food industry.

## 1. Introduction

Enterococci are Gram-positive lactic acid bacteria (LAB), which include pathogenic, spoilage, and commensal microorganisms. LAB are well suited to survival in the gastrointestinal tract of humans and animals as well as environments such as water, soil, and different types of food [1,2,3,4]. However, *Enterococcus* species are controversial because some clones are multidrug-resistant (MDR) and are a leading cause of nosocomial infections. Conversely, certain strains support the immune system as a probiotic (diet supplement or therapeutic application) [5]. Regardless, enterococci are generally considered secondary food contaminants, usually due to environmental and fecal contamination, and play a role in food spoilage [6], although many food products use enterococcal strains as a starter and-or in probiotic cultures in human healthcare and animal husbandry [7,8].

The alarming increase in MDR enterococci and their ability to acquire and transfer antimicrobial resistance (AMR) and virulence genes make their status in food uncertain, neither been Generally Recognised as Safe (GRAS) by the US Food and Drug Administration (FDA) (GRAS Notices (fda.gov)) nor included in the Qualified Presumption of Safe (QPS) list from the European Food Safety Authority (EFSA) (https://www.efsa.europa.eu/en/topics/topic/qps, accessed on 1 October 2022). On the other hand, the rising AMR in bacteria has attracted research interest in *Enterococcus* due to their capability to produce bacteriocins, bioactive substances active against a broad collection of spoiling and foodborne microbes [9].

The safety evaluation of enterococci remains challenging due to the limited knowledge of the type and combination of virulence factors necessary for their pathogenic potential. *Enterococcus faecalis (Efc)* and *Enterococcus faecium (Efm)* are the most frequent species responsible for human infections, and the worldwide dissemination of MDR enterococci is of great concern. Regardless, enterococci are actually not highly virulent organisms, and the success of *Efc* and *Efm* in hospital settings is primarily related to their survival capabilities in a hostile antimicrobial-rich environment. 

*Efm* represents the most important enterococci in food fermentation and spoilage and has also been reported as a probiotic for over two decades without any adverse effects [1]. On the other hand, *Efm* has rapidly evolved as a worldwide nosocomial pathogen [10], raising questions about its safety for use in foods or as probiotics. Considerable progress has been made in the last years to gain deeper insights into the genomic adaptability of *Efm* to various ecological niches. Thus, recent evidence suggests that the environment shapes phylogenetic relationships between dairy isolates and those from hospitals, communities, and animals [11,12,13,14]. Moreover, the accessory genome seems to play a key role in adapting different *Efm* populations; for example, a recent publication concluded that the plasmidomes rather than chromosomes are most informative for the source specificity of *Efm* [15]. Interestingly, the lack of genome defense mechanisms, such as CRISPR/cas, which provides immunity against bacteriophage infections and mobile genetic elements, has been related to the high genome plasticity in clinical isolates of *Efm* [16].

Early surveillance and risk assessment to establish microorganisms’ safety and/or efficacy when used in the food chain was primarily based on traditional microbiology. However, conventional microbiological methods require several labor-intense phenotypic and molecular testing, which can take several days to complete. Conversely, the dramatic reduction in cost and the increase in the quality of high-throughput sequencing (HTS) technologies make whole genome sequencing (WGS) feasible as a routine tool. Sequence-based methods, particularly Multi Locus Sequence Typing (MLST), have become the standard for epidemiological studies on enterococci. However, the emergence of WGS will supersede the MLST, allowing the implementation of core genome MLST (cgMLST) and the analysis of bacterial structure at an unsurpassed level of detail [17]. WGS offers a complete overview of an isolate and new possibilities for foodborne outbreak detection/investigation, source attribution, and hazard identification [18,19]. However, harmonized protocols and legislation are required for WGS to be used for microorganisms within the food chain. 

The recent availability of complete genomic sequences of *Efm* strains has paved the way for proteomic studies to elucidate their potential and safety concerns as probiotics. However, proteome complexity requires introducing analytical strategies based on innovation and sensitive mass spectrometric instruments. Recently, a new 4D-proteomics method coupled with parallel accumulation-serial fragmentation (PASEF) technology can reduce analysis time and lower sample requirements. Moreover, it improves protein identification, detection sensitivity, and data integrity [20]. Here, we propose a new proteomic workflow to identify and characterize potential *Efm* probiotic strains within 24 h, using a TimsTOF 4D proteomics instrument. This approach rapidly assesses *Efm*’s molecular mechanisms underlying probiotic actions and pathogenicity traits, making their use in food products safer and minimizing potential consumer risks.

## 2. Results

### 2.1. Genomic and Phylogenetic Analysis of Efm LHICA_28.4 and LHICA_40.4

The completeness of the assembled genomes, estimated by BUSCO-v5.4.3, was 99.5% for both LHICA 28.4 and LHICA 40.4. LHICA_28.4 belongs to cgMLST sequencing type-675, while LHICA 40.4 belongs to cgMLST sequencing type 1453. Based on the epidemiological data provided by PubMLST, ST-675 was isolated from a hospitalized patient in Spain in 2007 and a healthy non-hospitalized person in 2009. The ST-1453 was previously isolated from the environment in 2015. ResFinder-v4.1 did not provide additional information on the PGAP annotation, and VirulenceFinder-v2 identified two other virulence genes. Thus, LHICA 28.4 and LHICA 40.4 encoded 28 and 27 genes related to virulence and resistance to antimicrobials, heavy metals, and heat (disinfection) (Table 1). Regarding the in-silico search of bacteriocins, LHICA 28.4 encoded 14 different bacteriocin-like genes, whereas LHICA 40.4 encoded 7 (Table 2). CRISPRCasTyper did not identify CRISPR/Cas systems in the studied genomes, suggesting that the genomes lack defenses against mobile genetic elements and bacteriophages.

**Figure 1:**
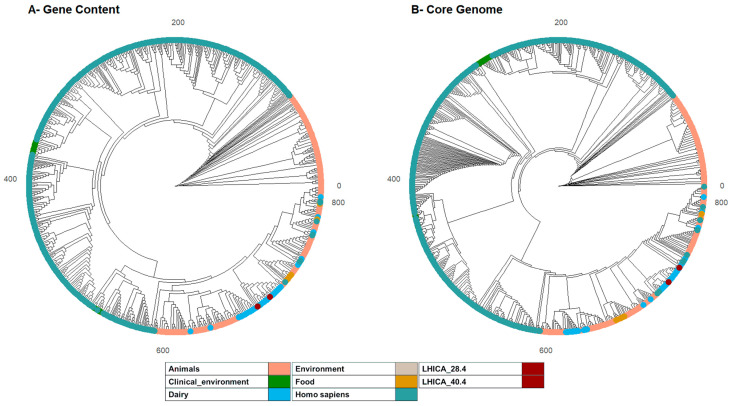
Pan-genome phylogenetic trees. The phylogenies of 812 *Efm* genomes are based on accessory gene content (**A**) and allelic variation in relaxed core genes (**B**). A different color indicates each *Efm* according to the source of isolation. (**A**) A FastTree phylogeny based on binary information of the presence and absence of 12,532 genes in the Efm pan-genome. (**B**) A RapidNJ phylogeny based on numbers of identical sequences (alleles) of single copy, relaxed, core genes present in ≥95% of *Efm* genomes.

The resulting pangenome contained 12,532 genes with a strict core genome of 1467 and a relaxed core genome of 1916. PEPPAN_parser was used to calculate two trees of the 812 genomes based on the presence or absence profiles of the pan genes (Figure 1A) and the allelic variation profiles of the relaxed core genes (Figure 1B). Both trees showed comparable tight clustering of genomes corresponding to different sources. LHICA 28.4 and LHICA 40.4 clustered with strains isolated from dairy products in both trees, which are non-human, neither animal-related *Efm* isolates.

### 2.2. LHICA_28.4 and LHICA_40.4 Proteomes

**Figure 2:**
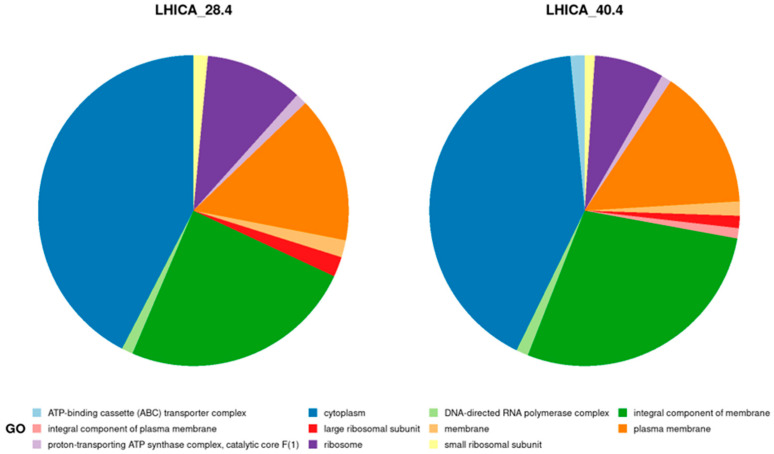
Cellular component classification in GO analysis for the proteome of LHICA 28.4 and LHICA 40.4. Describes the locations relative to cellular structures in which a gene product performs a function, either cellular compartments (e.g., mitochondrion) or stable macromolecular complexes of which they are parts (e.g., the ribosome).

TimsTOF Pro tandem mass spectrometer produced 167,232 (LHICA 28.4) and 166,761 (LHICA 40.4) spectra, identifying ~25% in both strains. A total of 7137 distinct peptides were identified for LHICA 28.4, corresponding to 1148 predicted protein-coding genes from its genome. Similar numbers were obtained for LHICA 40.4, with 6700 distinct peptides corresponding to 1117 predicted protein-coding genes. The abundance of the detected proteins seems to be related to the culture conditions, with higher counts for proteins related to carbohydrates and protein metabolism and fewer counts linked to dormancy and sporulation, virulence, disease, and defense traits (Figure S1). A total of 836 (LHICA 28.4) and 798 (LHICA 40.4) identified proteins were functionally annotated with KEGG, resulting in 22 complete KEGG pathway modules for both LHICA 28.4 and LHICA 40.4, mainly related to carbohydrate, energy, lipid, and nucleotide metabolism (Table S1). The GO functional annotation identified 307 and 430 proteins in LHICA 28.4 and LHICA 40.4, respectively, indicating the GO cellular component data that the extraction method obtained proteins from different compartments in the cell (Figure 2).

The proteome analysis quantified nine proteins related to antibiotic resistance, heavy metals, and heat; and two proteins related to virulence in LHICA 28.4. On the other hand, twelve resistance proteins and one gene associated with virulence traits were quantified in LHICA 40.4 (Table 1). The proteomic results could explain the previously reported resistance phenotype to cefazolin, oxacillin, erythromycin, and fosfomycin (LHICA 28.4). However, the resistance to clindamycin, sulfamide, and fosfomycin (LHICA 40.4) should be attributed to the presence of multidrug efflux pumps. Moreover, proteins conferring resistance to fluoroquinolones and aminoglycosides have been detected in LHICA 40.4. Finally, both LHICA 28.4 and LHICA 40.4 genomes encode for tetracycline resistance MFS efflux pumps, but neither of these proteins were quantified, nor were the strains reported resistant to tetracycline by Hosseini et al. [21].

The bacterial genomes of LHICA 28.4 and LHICA 40.4 encode several bacteriocin-like proteins, and their bactericidal activity against different pathogens has already been described (Table S2). Hosseini et al. previously reported Enterocin P’s presence in both isolated samples using a PCR screening to detect enterocins [21]. Our genome analysis shows that both isolates contain a high diversity set of bacteriocin-encoded genes. However, the proteomic analysis only quantified two bacteriocins from LHICA 28.4, a Mundticin KS and a Lactococcin 972 (Table 2). There may be various reasons why only two bacteriocins were detected. One possibility is that the low-molecular-weight bacteriocins (Table 2) were washed-out during the FASP digestion due to the 10 KDa molecular weight cut-off filter (MWCO).

## 3. Discussion

The controversial role of *Enterococcus* species in food safety requires more rapid and cost-effective methodologies for distinguishing between beneficial and potentially harmful strains. However, ideal methods should also identify the beneficial biotechnological properties of the bacterial strains, especially in the food sector. HTS technologies revolutionized the field of molecular biology by enabling large-scale WGS. Thus, today, portable sequencers can be applied to animal health and food safety to complement conventional surveillance strategies [22,23] Developments in bioinformatic analysis have accompanied the development of HTS. However, the main barriers to implementing HTS in the food industry are the higher degree of expertise in interpreting the results and the lack of international harmonization on bioinformatic analysis [24].

The success of HTS technologies for subtyping bacterial pathogens is undeniable. Thus, WGS has allowed for discrimination between beneficial and harmful *Efm* strains [25,26], and is increasingly used in public health laboratories for surveillance and outbreak investigation of foodborne pathogens. EFSA’s ambition seems to be implementing HTS as a gold standard to characterize microorganisms intentionally used in the food chain [19,27]. In this sense, EFSA permits using certain enterococcal strains as food additives and dietary supplements in animal nutrition based on a careful case-by-case assessment [28]. The EFSA guidance provides a methodology for distinguishing between safe and potentially harmful strains. According to this guidance, *enterococcal* strains shall be susceptible to ampicillin (MIC ≤ 2 mg/L) and lack IS16 (enhance genomic plasticity), hylEfm (putative glycoside hydrolase), and esp (an enterococcal surface protein involved in adhesion). However, the debate about the characterization of *Efm* has gained new prominence, with authors suggesting that the proposed criteria are not enough to discriminate *Efm* with the potential to cause human infections [29]. The strains from this study, LHICA 28.4 and LHICA 40.4, comply with those requirements. Indeed, the phylogenetic analysis clustered the strains among *Efm* isolated from dairy products, non-related to potential human pathogen strains. Therefore, LHICA 28.4 and LHICA 40.4 could be safely used as a probiotic in animal nutrition.

Metagenomics and WGS provide an overview of the complete inventory of genes recovered from complex samples or bacteria isolates. As a gene-centric approach, it is static and cannot fully reflect the temporal dynamics and functional activities of microbiomes or bacterial isolates. Moreover, genetic analysis is based on sequence homologies with genes in databases, limiting the risk and functionality evaluation. Therefore, knowing the genomic background of a beneficial bacteria, but not its functionality, does not offer any advance from a biotechnological point of view. To gain a more impactful understanding, proteomics must provide evidence of the actions or functions of the microorganism under different conditions. 

Here, we used a state-of-the-art shotgun proteomic method such as liquid chromatography coupled to a TimsTOF Pro instrument (Bruker Daltonics, Bremen, Germany) to characterize bacteriocin-producing *Efm* strains with the potential to be used as probiotics. Our current proteomic workflow does not just characterize the strains within only 24h, but it also increases by over 100 times the protein resolution compared with previous proteomic studies [30,31]. The resulting proteome explained the antibiotic resistance phenotypes that Hosseini et al. reported [21], and identified new resistance traits to fluoroquinolones and aminoglycosides. However, the analysis for bacteriocins shows the method’s limitation in purifying low-molecular-weight proteins. As such, results suggest that the protein extraction should use a lower MWCO filter in the FASP digestion.

The future development directions in using the metabolism characteristics of LAB in the food industry have been recently revised [32]. Thus, quantitative metabolic characterization of bacterial strains and microbiomes could offer expanded applications. On the one hand, LAB can degrade macromolecules and transform undesirable flavour substances. On the other hand, they can produce short-chain fatty acids, amines, bacteriocins, vitamins, and exopolysaccharides during their metabolism. Given these converse possibilities, the question for the food industry is not whether bacteria can perform a function but whether it is possible to control it efficiently. The present study quantified almost half of the proteins predicted in the bacterial genomes. Therefore, the current workflow could become a gold standard for determining and optimizing the bioengineering and biotechnology properties of LAB in the food industry.

## 4. Materials and Methods

### 4.1. Bacterial Strains and Whole-Genome

The bacteriocin-producing *Efm* strains used in this study belonged to the Laboratory of Food Hygiene and Control (LHICA, Lugo, Spain) collection at the University of Santiago de Compostela. Strains LHICA 28.4 and LHICA 40.4 were previously isolated from vacuum-packaged beef and evaluated for their probiotic aptitudes and the presence of enterocin-encoding genes [21]. Historical information about the strains is summarized in Table S2. Bacteria isolates were recovered from frozen stocks in de Man Rogosa Sharpe (MRS) broth (Oxoid, Ltd., London, UK) and plated on MRS agar (Oxoid, Ltd., London, UK). Pure cultures of each strain were harvested and resuspended in a tube with cryopreservative (Microbank™, Pro-Lab Diagnostics UK, Wirral, UK) and sent to MicrobesNG (Birmingham, UK) for genomic DNA extraction and sequencing. The strains’ DNA extraction and genome sequencing have been previously reported [33]. 

### 4.2. Sequence Analysis

The two genome assemblies were annotated using the NCBI Prokaryotic Genome Annotation Pipeline (PGAP) v5.3 [34], and genome completeness was assessed using Bandage-v0.8.1 and BUSCO-v5.4.3 with the *Lactobacilalles* database [35,36]. To further improve strains characterization, both assemblies were submitted to the web-based tools MLST-v2.0 [37], VirulenceFinder-v2.0 [38], and ResFinder-v4.1 [39] to identify the cgMLST and detect virulence, and resistance genes, respectively. The three abovementioned tools are operated and maintained by the Center for Genomic Epidemiology of the Denmark Technical University (http://www.genomicepidemiology.org/services, accessed on 1 October 2022). The CRISPR-Cas genes and arrays search was performed with the CRISPRCasTyper tool [40]. The *Efm* MLST database (http://pubmlst.org/efaecium/, accessed on 1 October 2022) was used to compare isolates with identical ST.

### 4.3. Phylogenetic Analysis

Relationships between the *Efm* strains from this study (LHICA_28.4 and LHICA_40.4) and the *Efm* lineage were explored using the Phylogeny Enhanced Pipeline for PAN-genome (PEPPAN) [41]. Briefly, 810 *Efm* genome assemblies were selected, attending to their source of isolation, from the National Center for Biotechnology Information (NCBI) Table S2. The genomes were then annotated using Prokka-v1.13 [42]. PEPPAN used the output files with default options. The source of isolation from the NCBI genomes was simplified in a new variable (mqb_source) (Table S3). Using this variable, PEPPAN was run with *Efm* isolated from *homo sapiens* (*n* = 485), animals (*n* = 235), dairy products (*n* = 56), clinical environments (*n* = 23), food (*n* = 7), and environmental samples (*n* = 4). The output from PEPPAN was parsed using PEPPAN_parser with ‘-m -t -c -a 95’ settings. PEPPAN_parser calculated two phylogenetic trees from the 812 genome assemblies, the first based on the presence or absence profiles of the entire *Efm* set of genes (pan genes) and the second on the allelic variation of core genes present in ≥95% of the genome assemblies. The topology of the first tree reflects similarities in pan-genome content, whereas the second tree reflects sequence similarities within core genes. Phylogenetic trees were visualized in R-v4.2.1 with the package ggtree-v3.4.2.

### 4.4. Proteomics

Sample preparation for proteomics was carried out as described earlier [43,44], starting from biomass collected from the LHICA 28.4 and LHICA 40.4 strains, plated on brain heart infusion media (BHI, Oxoid Ltd., Hampshire, UK) at 37 °C for 16 h. Proteins were obtained using an optimized phenol extraction protocol and digested with trypsin using filter-aided sample preparation (FASP). The purified peptide mixtures were then analyzed by reversed-phase liquid chromatography coupled to a TimsTOF™ Pro tandem mass spectrometer (Bruker Daltonik GmbH, Bremen, Germany), using a 120 min gradient. Proteome Discoverer Software (Thermo Fisher Scientific, Bremen, Germany, v1.4.1.14) converted the raw data into mascot generic files (MGFs). The MGFs were searched with the same parameters against a protein database using the search engines OMSSA [45] and X!Tandem [46] with the MetaProteomeAnalyzer-v3.1 (MPA) workflow [47], requiring at least one identified peptide for successful protein identification. Search results from the different search engines were merged after their individual scores were converted to uniform significance measures (q-values) [48], reflecting the minimum False Discovery Rate (FDR) for the identifications. Search parameters for the protein database searches were trypsin, one missed cleavage, monoisotopic mass, carbamidomethylation (cysteine) as fixed modification, oxidation (methionine) as variable modifications, ±10 ppm precursor and ±0.5 Da MS/MS fragment tolerance, 1^13^C and +2/+3 charged peptide ions. Results were controlled using a target-decoy strategy and a cut-off of 1% for FDR [49]. To characterize the proteomes, the initial annotations were complemented with functional information obtained from the integrated gene ontology database (GO) [50] and the Kyoto Encyclopedia of Genes and Genomes (KEGG) [51]. The protein quantification was obtained by label-free quantitative measures, including spectra count, normalized spectral abundance factor (NSAF), and the exponentially modified protein abundance index (emPAI). The visualization of functional information was performed in R-v4.2.1.

The protein databases were built with the predicted and annotated protein-coding genes from LHICA 28.4 and LHICA 40.4, which were downloaded from the GenBank assembly database with the accession numbers ASM2474191v1 (LHICA 28.4) and ASM2474193v1 (LHICA 40.4). SeqKit was used to remove potential duplicated sequences [52]. Thus, the final FASTA databases comprised 2548 and 2530 unique proteins for LHICA 28.4 and LHICA 40.4, respectively. VirulenceFinder-v2.0 annotated two additional virulence factors from both genomes, which were used for subsequent protein identification. The decoy databases were constructed by reversing the protein sequences from each database [53]. All mass spectrometry results were made publicly available by an upload to PRIDE [54], which could be accessed with the accession number PXD037241. More details on the resulting tables are included in Table S4.

## 5. Conclusions

Here, we used an end-to-end workflow for the rapid proteome analysis of bacterial strains of particular interest in the food industry. The work was successful, being able to identify almost half of the proteins predicted from the bacterial genomes of two bacteriocin-producing *Efm* strains. Furthermore, proteins conferring resistance to antibiotics, heavy metals, virulence factors, and bacteriocins were quantified, which provides safety guidance in assessing the bacterial strains. The obtained proteome matched the bacterial phenotype; however, slight modifications should be introduced in the extraction protocol to ensure that low-molecular-weight proteins are included in the analyses. The workflow speed and its higher resolution in protein identification make the flow ideal for expanding the bioengineering and biotechnology potential of beneficial metabolism characteristics of LAB in the food industry.

## Figures and Tables

**Table 1:** Genomic and proteomic characterization of LHICA 28.4 and LHICA 40.4. The table contains annotated genes related to resistance to antibiotics, heavy metals, and virulence factors in pale green. The quantified proteins against the predicted genes are marked with an “x” and highlighted in dark green. The RefSeq accession number and the description and role of the proteins are reported. In addition, label-free quantitative measures were included (SC: spectra count and emPAI).

RefSeq	LHICA 28.4	LHICA 40.4	Description	Role	SC	emPAI
WP_002299665.1	**X**	**X**	Multidrug efflux ABC transporter subunit EfrB	Multidrug resistance	1 | 7	0.05 | 0.2
WP_002289118.1	**X**	**X**	MBL fold metallo-hydrolase	Resistance to Beta-lactams	44 | 42	3.1 | 3.1
WP_002299607.1	**X**	**X**	MBL fold metallo-hydrolase	Resistance to Beta-lactams	16 | 22	1 | 1.6
WP_002319556.1	**X**		multidrug efflux MFS transporter	Multidrug resistance	1	0.1
WP_002293127.1	**X**		DNA gyrase subunit A	Resistance to fluoroquinolones	4	0.1
WP_002289238.1(*)	**X**		Collagen-binding MSCRAMM adhesin Acm	Virulence	36 | 24	1.6 | 1.1
WP_002316514.1(*)	**X**		Adhesin E. faecium	Virulence	9 | 16	0.8 | 2.16
WP_002287086.1			Multidrug efflux SMR transporter	Multidrug resistance		
WP_131774679.1			ABC-F type ribosomal protection protein Msr(C)	Resistance to macrolides		
WP_002286461.1			YihY/virulence factor BrkB family protein	Virulence		
WP_073461064.1	**X**		ATP-dependent Clp protease ATP-binding subunit	Heat resistance	1279	8.3
WP_002318484.1	**X**		Toxic anion resistance protein	Resistance to tellurite	77	3.5
WP_002294560.1	**X**		Copper homeostasis protein CutC	Resistance to copper	13	1.7
WP_010724442.1	**X**		FAD-containing oxidoreductase	Resistance to mercury	90	2.3
WP_114634998.1	**X**		DNA topoisomerase (ATP-hydrolyzing) subunit B	Resistance to fluoroquinolones	37	0.9
WP_002291207.1			Multidrug efflux MFS transporter EfmA	Multidrug resistance		
WP_002325116.1			Multidrug efflux MFS transporter	Multidrug resistance		
WP_002297435.1			Tetracycline resistance MFS efflux pump	Resistance to tetracycline		
WP_114635000.1			Tetronasin resistance protein	Resistance to tetronasin		
WP_002291784.1			CopY/TcrY family copper transport repressor	Resistance to copper		
WP_002299705.1			Cation diffusion facilitator family transporter	Resistance to cobalt-zinc-cadmium		
WP_002300950.1			Metalloregulator ArsR/SmtB family transcription factor	Resistance to cadmium		
WP_010723919.1			FosX/FosE/FosI family fosfomycin resistance hydrolase	Resistance to fosfomycin		
WP_258430621.1			CadD family cadmium resistance transporter	Resistance to cadmium		
WP_258430364.1			Virulence-associated E family protein	Virulence		
WP_002299664.1		**X**	Multidrug efflux ABC transporter subunit EfrA	Multidrug resistance	8	0.3
WP_002290958.1		**X**	Multidrug efflux MFS transporter	Multidrug resistance	1	0.1
WP_002293989.1		**X**	Aminoglycoside N-acetyltransferase AAC(6′)-Ii	Resistance to aminoglycoside	1	0.2
WP_002318652.1		**X**	Serine hydrolase	Resistance to Beta-lactams	6	0.4
WP_038809504.1		**X**	Multidrug efflux MFS transporter	Multidrug resistance	1	0.1
WP_002286766.1		**X**	Toxic anion resistance protein	Resistance to tellurite	75	3.5
WP_002288364.1		**X**	DNA topoisomerase (ATP-hydrolyzing) subunit B	Resistance to fluoroquinolones	33	0.8
WP_002300907.1		**X**	Heavy metal translocating P-type ATPase	Resistance to cobalt-zinc-cadmium	1	0.1
WP_002327434.1		**X**	FAD-containing oxidoreductase	Resistance to mercury	158	3.1
WP_002375871.1		**X**	Copper homeostasis protein CutC	Resistance to copper	11	1.3
WP_038809426.1		**X**	ATP-dependent Clp protease ATP-binding subunit	Heat resistance	1233	7.9
WP_002290060.1		**X**	Virulence factor B family protein	Virulence	5	0.2
WP_002311296.1			Multidrug MFS transporter	Multidrug resistance		
WP_038809639.1			Tetronasin resistance protein	Resistance to tetronasin		
WP_038809649.1			Tetracycline resistance MFS efflux pump	Resistance to tetracycline		
WP_002294855.1			CopY/TcrY family copper transport repressor	Resistance to copper		
WP_038809479.1			Cation diffusion facilitator family transporter	Resistance to cobalt-zinc-cadmium		

**Table 2:** Presence of bacteriocin-related proteins in LHICA 28.4 and LHICA 40.4. The table contains predicted bacteriocin-encoded genes in pale green. The quantified proteins against the predicted genes are marked with an “x” and highlighted in dark green. The RefSeq accession number and the description of the proteins are reported. In addition, label-free quantitative measures have been included (SC: spectra count and emPAI). Finally, the estimated molecular weight of the predicted bacteriocins was included.

RefSeq	LHICA 28.8	LHICA 40.4	Description	SC	emPAI	MW (KDa)
WP_002298900.1			Enterocin P precursor			5.80
WP_002318501.1			Bacteriocin secretion accessory protein			25.6
WP_002293180.1	X		Mundticin KS immunity protein	14	0.8	11
WP_002295295.1			Bacteriocin carnobacteriocin-A precursor			7.5
WP_002295575.1			Class IIb bacteriocin, lactobin A/cerein 7B family			6.2
WP_002307138.1			Blp family class II bacteriocin			6.9
WP_002323785.1	X		Lactococcin 972 family bacteriocin	1	0.2	16.6
WP_002338810.1			Enterocin P precursor			8
WP_002339189.1			Class II bacteriocin			6.9
WP_061343994.1			Bacteriocin immunity protein			13.7
WP_231369454.1			LsbB family leaderless bacteriocin			6.6
WP_038809770.1			Bacteriocin immunity protein			13.7
WP_072538874.1			Class II bacteriocin			6.4
WP_229210206.1			Enterocin B precursor			7.2
WP_229505516.1			LsbB family leaderless bacteriocin			6.5

## Data Availability

Not applicable.

## Supplementary Materials

The supporting information can be downloaded at: https://www.mdpi.com/article/10.3390/ijms232213830/s1.
