# Supplementary material for: Rapid Proteomic Characterization of Bacteriocin-Producing Enterococcus faecium Strains from Foodstuffs
Source: Int J Mol Sci. 2022 Nov 10;23(22):13830. doi: 10.3390/ijms232213830 (PMC9697693; doi:10.3390/ijms232213830)

Figure S1: Heatmap with the quantitative functional annotation of the LHICA 28.4 and LHICA 40.4 proteomes. Quantitative data was transformed into a logarithmic scale, and data were normalized by column to improve the visualization.

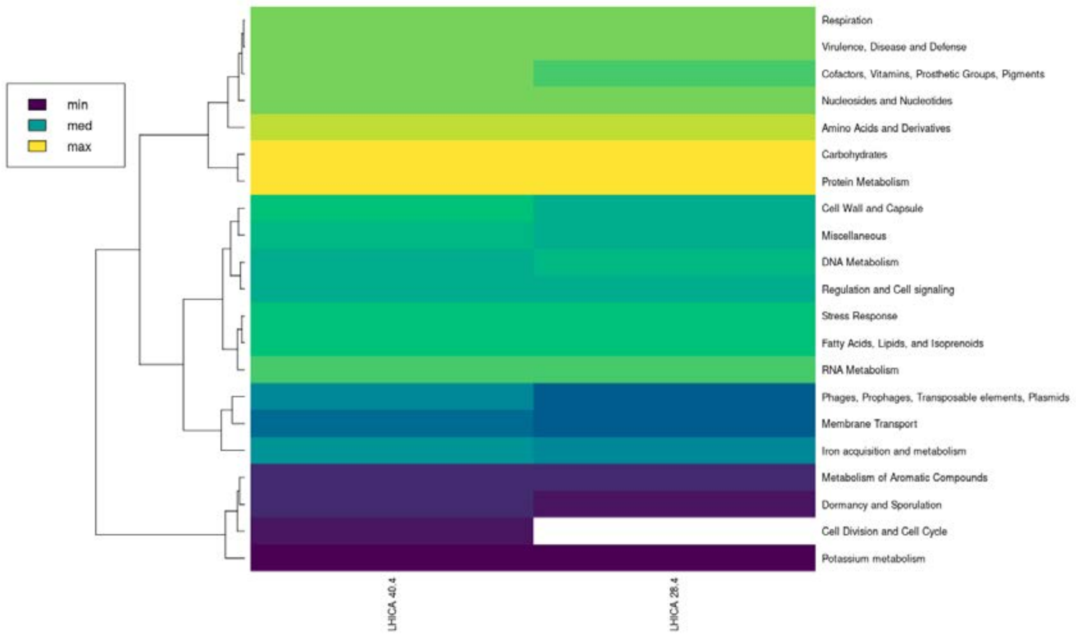

Supplement: Supplementary file 1 [file ijms-23-13830-s001.zip › supplementary_figure.pdf]
